# Strokes in Critically Ill COVID-19 Patients Diagnosed After Deep Sedation: A Single-Center Case Series

**DOI:** 10.7759/cureus.49993

**Published:** 2023-12-05

**Authors:** Gwen G Garrido-Bagayo, Jo Ann R Soliven

**Affiliations:** 1 Department of Neurology, The Medical City, Pasig City, PHL

**Keywords:** intensive & critical care, pandemic, ards, anticoagulation, sedation, hemorrhagic stroke, intracerebral hemorrhage, ischemic stroke, stroke, covid-19

## Abstract

In the Philippines, acute cerebrovascular disease is a common neurologic complication in coronavirus disease 2019 (COVID-19) patients. Because of sedation and limited neurological examination, the diagnosis of stroke in critically ill patients with COVID-19 may be delayed. This retrospective analysis was done on the medical records of adult patients with critical COVID-19 in 2021-2022 who were brought to a tertiary hospital in the Philippines, placed on mechanical ventilation, and later discovered to have had an ischemic or hemorrhagic stroke while under deep sedation. The study aimed to explore the delayed diagnosis of cerebrovascular disease clinically concealed by deep sedation and emphasizes the importance of a collaborative, multi-specialty approach to managing such patients.

There were nine patients with strokes discovered on imaging after deep sedation due to severe COVID-19 infection. The median age of the cases was 63 years, and 55.5% (n=5) were males. Three of the nine patients had an ischemic stroke with hemorrhagic conversion, three with ischemic infarction, and the other three had a primary intracerebral hemorrhage. This series shows a pattern of delayed diagnosis of cerebrovascular disease clinically concealed by deep sedation that was essential in managing acute respiratory distress syndrome in the background of severe COVID-19 infection before the emergence of severe acute respiratory syndrome coronavirus 2 (SARS‑CoV‑2) variants and the discovery of COVID-19 vaccines. The study demonstrates the significance of managing this unique population of patients in a collaborative and multi-specialty manner. With the continuing threat of SARS-CoV-2 and its variants, there is a need to strike a balance between the risks of ischemic and hemorrhagic stroke in COVID-19 infection and the care of this patient population.

## Introduction

According to the Philippine CORONA Study, acute cerebrovascular disease is the second most common neurologic complication among Filipino coronavirus disease 2019 (COVID-19) patients. Of those with acute cerebrovascular disease, 2.41% had acute infarctions, while 0.93% had hemorrhagic stroke [1].

Because of sedation and limited neurological examination, the diagnosis of stroke in critically ill COVID-19 patients may be delayed. Since patients with severe COVID-19 must be under deep sedation for days to weeks and receive therapeutic doses of anticoagulation, some studies recognize that some strokes of COVID-19 are diagnosed after prolonged sedation interruption. This retrospective case series aims to review patients with COVID-19 pneumonia placed on mechanical ventilation and deep sedation and identified to have an ischemic or hemorrhagic stroke during sedation weaning.

## Materials and methods

In this retrospective case series, we focused on adult patients (18 years old and above) meeting the criteria for "COVID-19 Critical" admitted in The Medical City, a tertiary training hospital in the Philippines, from January 1, 2021, to December 31, 2022. COVID-19 Critical patients in this study were defined as those with pneumonia and any of the following: facing impending respiratory failure, necessitating high-flow oxygen, non-invasive ventilation, or invasive ventilation; acute respiratory distress syndrome; and sepsis or shock [2].

Clinical suspicion, which included symptoms and signs that suggested cerebrovascular events, was the basis for the decision to refer patients to a neurologist and order neuroimaging. The attending physicians in charge of the patient's care made these clinical decisions. The classification and etiology of the stroke were determined based on the official interpretation of the neuroimaging studies, with a focus on the regions of infarction, hemorrhage, or other relevant findings. The attending neurologist, in conjunction with the neuroradiologist's report, contributed to this determination.

Inclusion and exclusion criteria

Inclusion criteria were patients with confirmed COVID-19, determined by a positive result in reverse transcriptase-polymerase chain reaction (RT-PCR) testing, who met the criteria for "COVID-19 Critical" based on the aforementioned definitions. Patients without evidence of ischemic or hemorrhagic stroke on neuroimaging were excluded from the analysis. Additionally, patients with neurological symptoms but no neurological imaging study and who were not referred to a neurologist were also excluded.

Data collection

Data collection encompassed various aspects.

Demographics

Data on demographics included patient age, gender, and any relevant demographic information.

Risk Factors

Information regarding underlying medical conditions and risk factors for stroke, including hypertension, diabetes, dyslipidemia, atrial fibrillation, and obesity, based on the Asian Pacific classification of BMI were collected.

Clinical Data

COVID-19 symptom development dates were identified based on comprehensive patient interviews, medical records, and documented onset of characteristic symptoms such as fever, cough, and respiratory distress. These sources were cross-referenced to ensure accuracy and reliability in determining the timeline of COVID-19 symptom manifestation. While reliance on patient recall and medical documentation introduces a degree of subjectivity, efforts were made to minimize inaccuracies through thorough data verification. Duration of mechanical ventilation, the onset of neurological deficits, clinical signs of stroke such as pupils' reactivity, sensorial changes, and other focal neurologic deficits were also reviewed and recorded.

Laboratory Data

D-dimer levels were recorded, along with indicators of inflammation, such as C-reactive protein, ferritin, and procalcitonin levels.

Clinical Outcomes

Patients' clinical outcomes, including whether they required continuous renal replacement therapy or developed bacteremia, were documented.

## Results

From January 1, 2021, to December 31, 2022, nine cases of stroke were diagnosed in patients classified as "COVID-19 Critical." These patients were referred to the Neurology service due to various new-onset neurologic deficits, as subsequently detailed, noted after they were weaned off from deep sedation. One case was referred for persistent impaired wakefulness, even though sedation weaning was underway, with only one sedating infusion remaining. Two patients were still deeply sedated when referred to the Neurology service: one due to a seizure and the other for prognostication. The clinical characteristics of these nine COVID-19 stroke patients are presented in Table 1.

**Table 1:** Clinical Characteristics of the Cases TOAST: Trial of Org 10172 in acute stroke treatment; COVID-19: coronavirus disease 2019; MCA: middle cerebral artery; PCA: posterior cerebral artery

Age (Years)	Sex	Lesion	Hemorrhagic Conversion	Distribution/ Location	TOAST Classification	Anticoagulation Dose	Anticoagulation Indication	Days Of COVID- 19 Symptoms Prior To Admission	Days of COVID-19 Symptoms Prior To Intubation	Days of COVID-19 Symptoms Before Stroke Discovery	Number of days of deep sedation prior to discovery of stroke	Days off sedation upon discovery of stroke
47	M	Ischemic Stroke	No	Multifocal, Bilateral	Cryptogenic	Prophylactic	Empiric	2	9	30	Still sedated with pentanyl and propofol	Still under midazolam and fentanyl infusion
61	M	Ischemic Stroke	Yes	Left, MCA	Cardioembolic	Prophylactic	Empiric	9	9	15	Still sedated with pentanyl and propofol	Still under midazolam and propofol infusion
60	F	Ischemic Stroke	Yes	Multifocal, Bilateral	Cardioembolic	Prophylactic	Empiric	3	3	14	Tapering down fentanyl and propofol continued	Still under fentanyl and propofol infusion
72	M	Ischemic Stroke	No	Left, MCA	Small Vessel Occlusion	Prophylactic	Empiric	14	19	34	14	On fentanyl infusion upon referral, off propofol infusion for three days
82	F	Hemorrhagic Stroke	Not applicable	Bilateral, multifocal	Not applicable	Prophylactic	Empiric	8	8	21	16	Off midazolam for 3 days, off fentanyl for 2 days
78	M	Ischemic Stroke	Yes	Right, MCA	Large Vessel Disease	Prophylactic	Empiric	8	8	16	10	On light sedation with dexmedetomidine
44	M	Hemorrhagic Stroke	Not applicable	Right temporal lobe	Not applicable	Prophylactic	Empiric	10	11	24	12	Still under midazolam and propofol infusion
63	F	Hemorrhagic Stroke	Not applicable	Multifocal, Bilateral	Not applicable	Prophylactic	Empiric	14	17	27	13	Still under midazolam and fentanyl infusion
88	F	Ischemic Stroke	No	Left, PCA	Cryptogenic	Prophylactic	Empiric	7	21	20	7	Still under midazolam infusion

The median age of these cases was 63 years, with 55.5% (n=5) being males. Each patient had at least one risk factor for stroke. The most common risk factors included hypertension and diabetes (five of nine patients), and four had dyslipidemia. Notably, two patients had atrial fibrillation, one of which was newly diagnosed. It is essential to consider that the new onset of atrial fibrillation can pose its own risk of embolic strokes. None of the patients had a history of coronary artery disease, and all were either obese or overweight based on the Asian Pacific classification of BMI.

The median hospital day of intubation was day three, and the median hospital day of stroke discovery was 18 days. The median duration of mechanical ventilation before the discovery of the stroke was 11 days. Eight patients were comatose without localizing neurological findings, while the other was drowsy and with unilateral weakness. Three patients had fixed and dilated pupils, one had anisocoria, and the rest had non-reactive or sluggishly reactive pupils that were isocoric. C-reactive protein, ferritin, and procalcitonin were elevated in the nine patients. D-dimers were also elevated, with a median peak value of 9010 ng/mL. All patients developed acute kidney injury during admission, and eight required continuous renal replacement therapy, with two refusing to undergo the procedure. Eight patients had bacteremia during their hospitalization, but only six had positive blood cultures at the imaging time.

All patients had severe acute respiratory distress syndrome (ARDS), two of which required neuromuscular blocking agents, and five required prone positioning. All patients were put under deep sedation to achieve a Richmond Agitation and Sedation Score of -4 to -5. The median days of deep sedation before stroke discovery was 12.5 days among the six patients already weaned from sedation. In contrast, three of the nine patients were still on sedation during stroke recovery. The details of sedation are given in Table 1.

Six patients had an ischemic stroke, three of whom had hemorrhagic conversion confined to regions of established infarctions. Three of the six ischemic infarct patients were diagnosed as radiologically acute, while the other three were subacute. Three were multifocal and bilateral ischemic infarcts, while the other three were in a vascular territory. According to the Trial of Org 10172 in Acute Stroke Treatment (TOAST) criteria [3], two patients were classified as cardioembolic, two were cryptogenic, and one was for small and large vessel occlusion. All ischemic infarct patients received empiric anticoagulation with a prophylactic dose of enoxaparin once a day prior to the discovery of the stroke. The median Glasgow Coma Scale at the time of stroke discovery was 3. Among the six of nine patients with a full National Institutes of Health Stroke Scale (NIHSS) assessed at the time of referral, the median NIHSS was 33, all of which had confounding effects of sedating medications.

The decision to administer empiric enoxaparin aligns with the Philippines’ Interim Guidance on the Clinical Management of Adult Patients with Suspected or Confirmed COVID-19 Infection [4]. These guidelines recommend anticoagulation as part of the therapeutic approach for COVID-19 patients to mitigate the risk of thrombotic events. While the use of empiric enoxaparin was in line with national recommendations, it is an aspect that warrants further investigation and consideration in future studies. Three of the nine patients in this series were found to have primarily acute intracerebral hemorrhages. Of these, two patients had multicompartment hemorrhage with intraventricular extension, mass effect, and midline shift, while the third patient had only a small intracranial hemorrhage in the right temporal lobe. All three patients received empiric anticoagulation with a prophylactic dose of enoxaparin before the discovery of the stroke. All nine patients in this case series expired during their hospital stay. 

## Discussion

In concordance with the findings of Bruce et al.'s study conducted in a New York City academic tertiary care referral center [5], this retrospective case series highlights the diagnostic challenges in identifying strokes in critically ill COVID-19 patients. The constraint imposed by deep sedation often results in delayed recognition of neurologic deficits, emphasizing the need for nuanced approaches to stroke management in this population.

The intricate interplay between SARS-CoV-2 and angiotensin-converting enzyme 2 (ACE2) receptors, triggering hypercoagulability and thrombophilia, elucidates the heightened risk of acute ischemic stroke [6]. However, the proposed mechanism's clarity in explaining strokes, particularly those attributed to small vessel disease, remains an open question. While it may robustly elucidate large vessel strokes or embolic etiologies, its applicability to small vessel strokes warrants further exploration. On the other hand, although intracerebral hemorrhage is a rare complication of COVID-19, it is life-threatening, nonetheless. A proposed mechanism of intracerebral hemorrhage (ICH) in COVID-19 infection is associated with arterial hypertension induced by the binding of SARS-CoV-2 to ACE2 receptors and thrombocytopenia [7,8].

Applying the lessons from Bruce et al.'s study [5] to our academic tertiary care center in the Philippines, we emphasize the crucial role of early stroke diagnosis. Although thrombolytic interventions may be limited due to uncertain ictus times, prompt diagnosis remains pivotal for investigating stroke etiology and optimizing secondary stroke prevention. The decision regarding anticoagulation continuation or cessation is equally vital, considering its implications for prognosis and patient management.

Delving into treatment optimization for critically ill COVID-19 patients, reevaluating the utility of regular sedation holidays becomes imperative. Balancing potential benefits with the challenges and risks in our unique patient population is a critical consideration [9,10]. Introducing surveillance cranial CT scans, despite logistical complexities, emerges as a valuable tool for gaining insights into the neurologic status of these patients.

The empiric use of anticoagulation, guided by Philippine guidelines on the clinical management of adult COVID-19 patients, aligns with existing studies but prompts a call for a more comprehensive risk-benefit assessment in this particular patient subset. As we navigate these considerations, it is crucial to acknowledge the study's limitations. Being a retrospective chart review, biases inherent to such designs must be recognized, emphasizing the need for a cautious interpretation of the findings. Additionally, relying on TOAST classification for stroke etiology introduces complexities in the multifactorial landscape of COVID-19-related strokes.

## Conclusions

The significance of the current study lies not only in its specific findings but also in the lessons learned from managing strokes in this challenging subset of patients. Recognizing the limitations of a retrospective review, our study prompts a deeper exploration of stroke risks in critically ill, deeply sedated COVID-19 patients. The unique clinical scenario presented in our study underscores the importance of nuanced approaches to stroke detection, emphasizing risk factors pertinent to this population.

While this small-scale case series sheds light on the challenges of detecting cerebrovascular events in deeply sedated, critically ill COVID-19 patients, it is crucial to acknowledge the need for further studies to comprehensively determine the stroke risk in this specific population. Our findings, while not exhaustive, mirror the experiences of a major academic hospital in the Philippine capital during the height of the pre-vaccine COVID-19 pandemic. This case series serves as a stepping stone, urging the scientific community to delve deeper into the intricate relationship between COVID-19, sedation, and stroke. Our hope is that these preliminary insights will fuel further investigations, ultimately contributing to a more nuanced understanding of cerebrovascular events in critically ill COVID-19 patients and guiding future clinical strategies.
